# Translating research into practice: outcomes from the Healthy Living after Cancer partnership project

**DOI:** 10.1186/s12885-020-07454-4

**Published:** 2020-10-06

**Authors:** Elizabeth G. Eakin, Marina M. Reeves, Ana D. Goode, Elisabeth A. H. Winkler, Janette L. Vardy, Frances Boyle, Marion R. Haas, Janet E. Hiller, Gita D. Mishra, Michael Jefford, Bogda Koczwara, Christobel M. Saunders, Kathy Chapman, Liz Hing, Anna G. Boltong, Katherine Lane, Polly Baldwin, Lesley Millar, Sandy McKiernan, Wendy Demark-Wahnefried, Kerry S. Courneya, Jennifer Job, Natasha Reid, Erin Robson, Nicole Moretto, Louisa Gordon, Sandra C. Hayes

**Affiliations:** 1 The University of Queensland, Brisbane, QLD Australia; 2 School of Public Health, Faculty of Medicine, The University of Queensland, Herston Road, Herston, QLD 4006 Australia; 3 University of Sydney, Sydney, NSW Australia; 4 Mater Hospital, Sydney, NSW Australia; 5 University of Technology Sydney, Sydney, NSW Australia; 6 Swinburne University of Technology, Melbourne, VIC Australia; 7 Peter MacCallum Cancer Centre, Melbourne, VIC Australia; 8 University of Melbourne, Carlton, VIC Australia; 9 Flinders University, Adelaide, SA Australia; 10 University of Western Australia, Perth, WA Australia; 11 University of Newcastle, Newcastle, NSW Australia; 12 Cancer Council New South Wales, Woolloomooloo, Australia; 13 Victorian Comprehensive Cancer Centre, Parkville, VIC Australia; 14 Cancer Council Victoria, Melbourne, VIC Australia; 15 Cancer Council South Australia, Adelaide, SA Australia; 16 Cancer Council Western Australia, Perth, WA Australia; 17 University of Alabama at Birmingham, Birmingham, USA; 18 University of Alberta, Edmonton, Canada; 19 QIMR Berghofer Medical Research Institute, Brisbane, Queensland Australia; 20 Griffith University, Menzies Health Institute Queensland, Brisbane, QLD Australia

**Keywords:** Lifestyle intervention, Cancer survivors, Dissemination and implementation study, Physical activity, Nutrition, Healthy weight

## Abstract

**Background:**

Healthy Living after Cancer (HLaC) was a national dissemination and implementation study of an evidence-based lifestyle intervention for cancer survivors. The program was imbedded into existing telephone cancer information and support services delivered by Australian state-based Cancer Councils (CC). We report here the reach, effectiveness, adoption, implementation, and maintenance of the program.

**Methods:**

In this phase IV study (single-group, pre-post design) participants - survivors of any type of cancer, following treatment with curative intent - received up to 12 nurse/allied health professional-led telephone health coaching calls over 6 months. Intervention delivery was grounded in motivational interviewing, with emphasis on evidence-based behaviour change strategies. Using the RE-AIM evaluation framework, primary outcomes were reach, indicators of program adoption, implementation, costs and maintenance. Secondary (effectiveness) outcomes were participant-reported anthropometric, behavioural and psychosocial variables including: weight; physical activity; dietary intake; quality-of-life; treatment side-effects; distress; and fear of cancer recurrence and participant satisfaction. Changes were evaluated using linear mixed models, including terms for timepoint (0/6 months), strata (Cancer Council), and timepoint x strata.

**Results:**

Four of 5 CCs approached participated in the study. In total, 1183 cancer survivors were referred (mostly via calls to the Cancer Council telephone information service). Of these, 90.4% were eligible and 88.7% (*n* = 791) of those eligible consented to participate. Retention rate was 63.4%. Participants were mostly female (88%), aged 57 years and were overweight (BMI = 28.8 ± 6.5 kg/m2). Improvements in all participant-reported outcomes (standardised effect sizes of 0.1 to 0.6) were observed (*p* < 0.001). The program delivery costs were on average AU$427 (US$296) per referred cancer survivor.

**Conclusions:**

This telephone-delivered lifestyle intervention, which was feasibly implemented by Cancer Councils, led to meaningful and statistically significant improvements in cancer survivors’ health and quality-of-life at a relatively low cost.

**Trial registration:**

Australian and New Zealand Clinical Trials Registry (ANZCTR) - ACTRN12615000882527 (registered on 24/08/2015).

## Background

The number of cancer survivors is rapidly increasing worldwide [1]. This is largely due to improved screening and treatments leading to increased survival rates for the majority of cancers. In Australia, survival rates continue to improve, with 5-year survival for all cancers at 69% for the period 2011–2015 (up from 50% in 1986–1990) and over 90% for some cancers, such as prostate (95%) and breast cancer (91%) [2]. While a highly positive trend, longer cancer survivorship also results in higher risk of certain adverse outcomes, including cancer recurrence, second primaries, persistent side-effects of treatment, functional decline and co-morbid chronic conditions, such as cardiovascular disease and type 2 diabetes [3–6]. Engagement in regular physical activity, improvement in diet and keeping within a healthy weight range are recognised as evidence-based methods of mitigating these long-term risks and are recommended by most national cancer organisations [7–9]. These recommendations are supported by a strong body of evidence showing they lead to improved survivorship outcomes; yet, adherence to these recommendations is poor [10]. More than half of cancer survivors are overweight or obese, more than half do not meet physical activity recommendations, and two-thirds do not meet dietary guidelines [11]. Following cancer treatment, declines in activity and weight gain are also common [12–14]. While most cancer survivors desire guidance regarding healthy lifestyles [15, 16], and there is increased recognition of the importance of improving healthy lifestyle behaviours in models of survivorship care [17], cancer care does not routinely include such assistance [15, 18, 19].

A large body of research, as summarised in systematic reviews and meta-analyses [20–22] has demonstrated that lifestyle interventions are efficacious in improving a range of behavioural and clinical outcomes in survivors of various cancers. The evidence is strongest in women with breast cancer and for physical activity interventions [23], but there is also evidence for the benefits accrued from dietary interventions [24] and for weight loss interventions [25]. These interventions have also been shown to have a maintenance effect at least 3 months post-intervention completion [26]. Given the strength of evidence, the focus of more recent research has turned toward the evaluation of broad-reach or distance intervention modalities, particularly those that are telephone-based, in order to understand if this delivery mode has the potential for greater reach than face-to-face interventions, while maintaining effectiveness [27–29]. A systematic review of 27 trials of broad-reach lifestyle interventions among cancer survivors, where 22 were based on telephone delivery, indeed found evidence for improvements in lifestyle behaviours and weight loss across cancer survivor groups [30]. Another study, which compared the effectiveness of a telephone versus face-to-face delivered intervention for achieving improvements in fitness and quality of life in women treated for breast cancer, found the two modalities did not differ in effectiveness [31, 32]. Importantly, the study concluded that telephone-based interventions are considered suitable for reaching women living in regional and rural Australia [33]. This large body of evidence supports the present study.

The Healthy Living after Cancer (HLaC) Partnership Project is a dissemination and implementation trial evaluating the effect of a 6-month, telephone-based lifestyle intervention for cancer survivors delivered by four Australian state-based Cancer Councils (non-government organisations) as part of their Cancer Information and Support Service. The Cancer Councils were highly aligned partners for this work in that: they each had a mandate to provide survivorship services on a state-wide basis, noting that 30% of Australians with cancer live outside of metropolitan areas [34]; and they had the telephonic infrastructure and staff to implement the project. The RE-AIM framework (reach, effectiveness, adoption, implementation, maintenance) [35] is used for evaluation as its emphasis on indicators of both internal validity (effectiveness) and external validity (implementation) is aligned to the dissemination context of the study. We report on primary outcomes (program adoption, reach, implementation, costs and maintenance) and secondary (effectiveness) outcomes including anthropometric, behavioural and psychosocial changes from pre – (baseline) to post - (6 month) program and participant satisfaction.

## Methods

### Study design

Detailed methods for the HLaC study have been previously reported [36]. The study received ethical approval from: The University of Queensland, Cancer Council Victoria (on behalf of Cancer Councils Victoria and South Australia), Cancer Council New South Wales, and the University of Western Australia (on behalf of Cancer Council Western Australia).

### Participants and recruitment

**Fig. 1:**
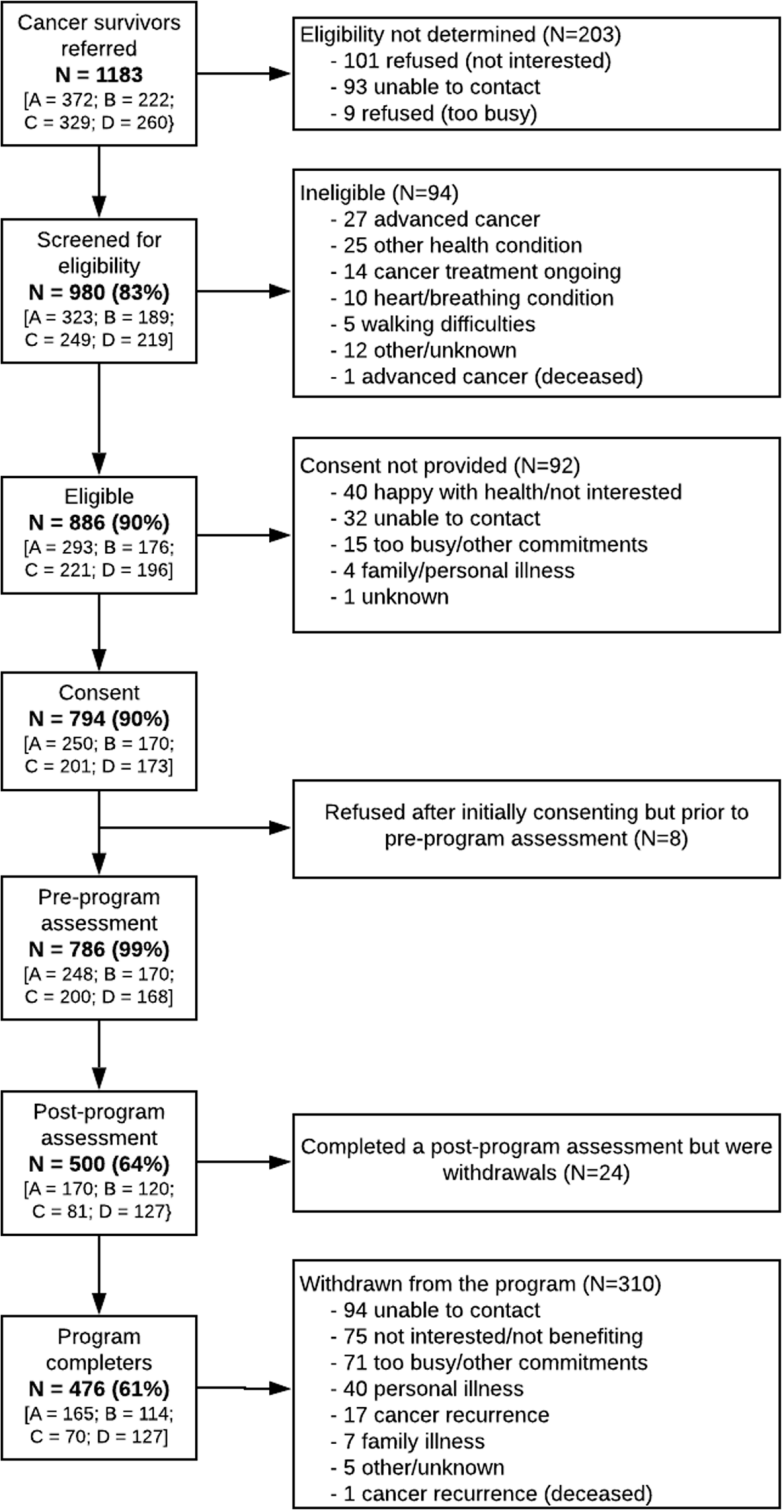
HLaC participant flowchart

Participants were referred to the program between June 2015 and September 2018 (see Fig. 1). Inclusion criteria were designed to be as broad as possible, thereby maximising the diversity of survivors able to take part safely. The eligibility criteria were:

- ◦ Adults (18+ years); diagnosed with a cancer of any type that was localised, non-metastatic and treated with curative intent;
- ◦ Completed primary treatment (ongoing hormonal treatment or trastuzumab was permitted);
- ◦ No contraindications to engaging in unsupervised physical activity, including but not limited to: active heart disease, breathing problems, planned knee or hip replacement, pregnant or intending to become pregnant in the next 6 months;
- ◦ No cognitive or mental health impairments that would hinder program participation;
- ◦ Sufficiently proficient in the English language to meaningfully participate in the program;
- ◦ Wanting support for healthy living via physical activity and healthy eating and willing to make a six-month commitment to program participation.

Participant eligibility was self-reported to Cancer Council staff who conducted screening over the telephone using a recruitment and screening script (refer to Table 1 in published protocol paper [36]). In cases where eligibility was uncertain, clearance was sought from the participants treating clinician.

**Table 1:** Baseline Characteristics of Healthy Living After Cancer (HLaC) participants overall and by Cancer Council

Overall	Overall		A		B		C		D		p ^**a**^
	n	Summary	n	Summary	n	Summary	n	Summary	n	Summary	
Age (M ± SD)	786	57.52 ± 11.44	248	57.43 ± 11.74	170	58.26 ± 10.52	200	55.34 ± 11.63	168	59.52 ± 11.29	**0.004**
Male	94	12.0%	32	12.9%	16	9.4%	29	14.5%	17	10.1%	0.384
Female	692	88.0%	216	87.1%	154	90.6%	171	85.5%	151	89.9%	
Ethnicity											0.006 ^b^
Aboriginal or Torres Strait Islander	7	0.9%	5	2.0%	1	0.6%	1	0.5%	0	0.0%	
Asian	39	5.0%	14	5.6%	3	1.8%	13	6.5%	9	5.4%	
Caucasian or white	705	89.7%	212	85.5%	162	95.3%	179	89.50%	152	90.5%	
Middle Eastern	9	1.1%	3	1.2%	2	1.2%	3	1.5%	1	0.6%	
Pacific Islander	7	0.9%	5	2.0%	0	0.0%	0	0.0%	2	1.2%	
South American	7	0.9%	5	2.0%	0	0.0%	1	0.5%	1	0.6%	
Other	10	1.3%	3	1.2%	1	0.6%	3	1.5%	3	1.8%	
Not reported	2	0.3%	1	0.4%	1	0.6%	0	0.0%	0	0.0%	
Comorbidities											
0	160	20.4%	61	24.6%	29	17.1%	44	22.0%	26	15.5%	**0.005**
1	189	24.0%	68	27.4%	38	22.4%	48	24.0%	35	20.8%	
2	160	20.4%	46	18.5%	41	24.1%	40	20.0%	33	19.6%	
3	123	15.6%	28	11.3%	30	17.6%	34	17.0%	31	18.5%	
≥ 4	154	19.6%	45	18.1%	32	18.8%	34	17.0%	43	25.6%	
Education											
< High School	115	14.6%	34	13.7%	33	19.4%	31	15.4%	17	10.1%	**0.004**
High School	71	9.0%	16	6.5%	24	14.1%	19	9.5%	12	7.1%	
TAFE / Technical	267	34.0%	72	29.0%	57	33.5%	69	34.5%	69	41.1%	
University	333	42.4%	126	50.8%	56	32.9%	81	40.5%	70	41.7%	
BMI, kg/m^2^ M ± SD	786	28.81 ± 6.51	248	27.67 ± 6.47	170	30.14 ± 6.52	200	29.05 ± 6.28	168	28.85 ± 6.59	**0.002**
Employment											
Full time	149	19.0%	48	19.4%	25	14.7%	44	22.0%	32	19.0%	0.261
Part-time / Casual	231	29.4%	69	27.8%	50	29.4%	61	30.5%	51	30.4%	
Self employed	13	1.7%	4	1.6%	1	0.6%	5	2.5%	3	1.8%	
Home duties	62	7.9%	21	8.5%	14	8.2%	15	7.5%	12	7.1%	
Retired	164	20.9%	57	23.0%	29	17.1%	34	17.0%	44	26.2%	
Unable to work	104	13.2%	32	12.9%	34	20.0%	24	12.0%	14	8.3%	
Other not working	63	8.0%	17	6.9%	17	10.0%	17	8.5%	12	7.1%	
Alcohol (standard drinks/ week) ^c^											
0	390	49.6%	120	48.4%	81	47.6%	101	50.5%	88	52.4%	0.867
> 0 to < 21	370	47.1%	118	47.6%	82	48.2%	96	48.0%	74	44.0%	
21 to < 35	21	2.7%	8	3.2%	6	3.5%	2	1.0%	5	3.0%	
≥ 35	5	0.6%	2	0.8%	1	0.6%	1	0.5%	1	0.6%	
Marital status											
Married / living together	501	63.7%	160	64.5%	109	64.1%	120	60.0%	112	66.7%	0.681
Divorced	124	15.8%	34	13.7%	30	17.6%	32	16.0%	28	16.7%	
Separated	31	3.9%	14	5.6%	4	2.4%	7	3.5%	6	3.6%	
Widowed	34	4.3%	13	5.2%	6	3.5%	10	5.0%	5	3.0%	
Never married	96	12.2%	27	10.9%	21	12.4%	31	15.5%	17	10.1%	
IRSAD											
Bottom 30% of state	133	17.1%	35	14.1%	35	21.2%	40	20.0%	23	13.9%	0.268
Middle 40% of state	299	38.5%	93	38.0%	66	40.0%	77	38.5%	63	51.8%	
Top 30% of state	344	44.3%	117	47.8%	64	38.8%	83	41.5%	80	48.2%	
Missing	10	–	3	–	5	–	0	–	2	–	
Geographic location											
Major City	594	76.3%	198	80.8%	112	67.1%	149	74.5%	135	80.4%	**0.005**
Elsewhere	184	23.7%	47	19.2%	55	32.9%	51	25.5%	31	18.7%	
Missing	8	–	3	–	3	–	0	–	2	–	
Years since diagnosis: M ± SD, Median (min, max)	786	1.91 ± 3.02	248	2.11 ± 2.72	170	1.86 ± 2.98	200	1.70 ± 1.95	168	1.92 ± 4.28	0.316
		1 (0, 54)		1 (0, 20)		1 (0, 32)		1 (0, 15)		1 (0, 54)	
Cancer type											
Breast	484	61.6%	143	57.7%	113	66.5%	114	56.7%	114	67.9%	0.113 ^b^
Cervical	12	1.5%	6	2.4%	1	0.6%	4	2.0%	1	0.6%	
Colorectal / bowel	71	9.0%	18	7.3%	16	9.4%	22	11.0%	15	8.9%	
Kidney	7	0.9%	2	0.8%	1	0.6%	4	2.0%	0	0.0%	
Lung	7	0.9%	3	1.2%	0	0.0%	1	0.5%	3	1.8%	
Lymphoma	66	8.4%	22	8.9%	15	8.8%	17	8.5%	12	7.1%	
Melanoma	2	0.3%	0	0.0%	0	0.0%	1	0.5%	1	0.6%	
Prostate	30	3.8%	11	4.4%	7	4.1%	5	2.5%	7	4.2%	
Stomach	2	0.3%	0	0.0%	0	0.0%	2	1.0%	0	0.0%	
Testicular	3	0.4%	0	0.0%	1	0.6%	2	1.0%	0	0.0%	
Ovarian	19	2.4%	10	4.0%	4	2.4%	4	2.0%	1	0.6%	
Uterine	15	1.9%	2	0.8%	2	1.2%	9	4.5%	2	1.2%	
Endometrial	10	1.3%	5	2.0%	2	1.2%	2	1.0%	1	0.6%	
Leukaemia	11	1.4%	5	2.0%	4	2.4%	2	1.0%	0	0.0%	
Other	47	6.0%	21	8.5%	4	2.4%	11	5.5%	11	6.5%	
***Treatment***											
Surgery	686	87.3%	208	83.9%	147	86.5%	178	89.0%	153	91.1%	0.140
Chemotherapy	531	67.6%	166	66.9%	123	72.4%	128	64.0%	114	67.9%	0.389
Radiotherapy	469	59.7%	155	62.5%	103	60.6%	119	59.5%	92	54.8%	0.465
Hormone therapy	343	43.6%	105	42.3%	62	36.5%	92	46.0%	84	50.0%	0.073
trastuzumab	75	9.5%	19	7.7%	22	12.9%	13	6.5%	21	12.5%	0.069

*IRSAD* index of relative socioeconomic advantage and disadvantage, *BMI* body mass index

^a^ p for difference between cancer council by ANOVA (M ± SD) or chi-square test (%)

^b^ due to insufficient frequencies chi-square test based on collapsed categories: Caucasian / other; breast / colorectal or bowel / lymphoma / prostate / other

^c^ Measured per week: cut-offs based on daily thresholds of none, > 0 to < 3/day, 3 to < 5 / day, and > =5 /day)

### Healthy living after Cancer program

A detailed description of the HLaC program is provided elsewhere [36]. Briefly, the intervention was based on Social Cognitive Theory constructs including self-efficacy, social support and outcome expectancies [37] and guided by techniques of motivational interviewing [38] and health behaviour coaching [39]. The program was aimed at increasing physical activity, promoting healthy eating, and assisting with moderate weight loss (if indicated), consistent with current evidence and guidelines for nutrition and physical activity in cancer survivors [7–9]. Participants were encouraged to consider making changes in all target areas (physical activity, diet, weight loss), but were able to choose to focus on one, two or all three domains. They received up to 12 coaching calls over the six-month program and a Participant Workbook [36], and were guided to develop skills in goal setting, self-monitoring, problem solving, identifying social support, stimulus control, positive self-talk and self-reward [40]. Cancer Council nurses/allied health professionals were trained by lead study investigators in the intervention protocol during a two-day workshop and provided with a training manual containing detailed call-by-call scripts and checklists. A lead nurse/health professional at each Cancer Council was designated to train new staff in a train-the-trainer approach. To address fidelity of intervention delivery during the first 2 years of the program, intervention calls were voice recorded approximately monthly for feedback and monthly case teleconferences were held.

### Data collection

Data were collected at baseline and six-months (post-program; primary endpoint) by study-trained Cancer Council staff using validated questionnaires [36]. Monthly database reports submitted by Cancer Council staff to the research team were used to monitor protocol implementation and data quality.

### Primary outcomes

Primary outcomes were mapped to the RE-AIM framework [35].

Reach: number of referrals and referral source; program uptake, participant characteristics.

Implementation: study retention, program completion and call delivery (number and duration of calls) and adverse events.

A serious adverse events protocol required Cancer Council staff to report these to study investigators within 24 h. Events were classified by investigators as severe/undesirable (significant symptoms requiring hospitalisation or invasive intervention), life threatening / disabling (acute, life-threatening complication or consequences), or fatal (death related to serious adverse event).

Costs: Costs of program delivery are reported with the methodology described in Additional file 1.

Maintenance: is reported as the number of Cancer Councils continuing or discontinuing HLaC following the end of the study.

### Secondary outcomes (effectiveness)

Secondary outcomes were all self-reported and mostly assessed using measures validated for use with cancer populations [36].

Anthropometric measures were weight (kg and body mass index BMI [weight in kg / height in m^2^]) and waist circumference (cm).

Time spent in moderate-vigorous physical activity (MVPA; min/week) was collected using the Active Australia Survey [41] using standard scoring (i.e., truncating individual items at 840 min and the total score [walking time, other moderate activity time excluding gardening, and 2 x vigorous activity time] at 1680 min). Sitting time (hours/day) was assessed using the Active Australia Survey [41] weekday sitting item.

Daily serves of fruit and vegetables were assessed using National Health Survey items [42].

A Fat Index and Fibre Index (scored 1–5, with higher values indicating healthier behaviours) was obtained using the validated 20-item Fat and Fibre Behaviour Questionnaire [43].

Quality of life was assessed using the Physical and Mental Components Scores of the Short-Form Health Survey SF-12, V1 with Australian weightings [44], for which higher values indicate better quality of life. To minimise unnecessary data loss and potential bias from non-reporting of a small number of items, up to three missing items, replaced by their group mean, were permitted [45].

Cancer and treatment-related symptoms and side effects were assessed using the Symptom Severity and Symptom Interference scores of the MD Anderson Symptom Inventory [46]. Ten core symptoms were assessed: fatigue, sleep disturbance, distress, shortness of breath, poor memory, poor appetite, drowsiness, sadness and numbness; an average was then taken. Higher scores (0–10) indicate greater severity or interference.

Fear of cancer recurrence was assessed using the 4-item Concerns about Recurrence Questionnaire [47]. Scores were calculated by summing four items, after first converting the 0–100% likelihood of recurrence item to the same 0–10 scale as the remaining items. Higher scores reflect greater fear, worry or concern. Missing items (nearly always the likelihood of recurrence question) were replaced with the mean of the participant’s other items.

Distress level and impact of distress were assessed from a modified 2-item distress thermometer [48] which asked participants to rate their level of distress over the past week from 0 (least distress) to 10 (most distress) and the impact of that distress on doing day-to-day activities from 0 (no impact) to 10 (highest impact).

Participant satisfaction was assessed with regard to the program overall, the coaching calls, and the program workbook, using a 5-point Likert scale.

### Sample size

As described in detail elsewhere [36], the sample size was chosen a priori to provide at least 90% power (with two-tailed significance of *p* < 0.001) to detect minimum differences of interest in body weight (2 kg), moderate-vigorous physical activity (60 min/week), fruit (0.5 serves), vegetables (0.5 serves), and physical and mental quality of life (3 units).

### Statistical analyses

Analyses were performed in IBM SPSS Statistics for Windows 24.0 (IBM Corporation, Armonk NY USA). Statistical significance was set at two-tailed *p* < 0.001. Process outcomes were reported using descriptive statistics as means and standard deviations or number and percentages as appropriate. Changes over time in patient-reported outcomes were analysed using mixed models, including all available data from all participants (*n* = 753 to 786 at baseline and *n* = 461 to 500 at post intervention). Models included effects of time (baseline / post intervention), strata (Cancer Council: A / B / C / D), and time x Cancer Council interaction. Changes are reported overall (pooled) and within each strata, based on comparisons of marginal means. To assess the sensitivity of conclusions to missing data, results are also reported adjusted for predictors of missing data, and using multiple imputation (m = 50 imputations). Dropout accounted for the vast majority of missing data. For most outcomes, predictors of missing data were treated as variables associated with dropout at *p* < 0.2 with predictors of missing data assessed separately for waist circumference, fibre index scores, and fear of cancer recurrence, which had approximately 5–10% item missing data. Imputation was by the fully conditional specification method, with predictive mean matching. Imputation models contained the variables in the analytic models, predictors of missing data, plus auxiliary variables that may help predict the missing outcomes (variables associated with the outcome at p < 0.2) (see Additional file 2). A per protocol analysis was also performed, evaluating changes in patient-reported outcomes among program completers (i.e., those who received ≥4 intervention calls and underwent the post-program evaluation).

## Results

### Primary outcomes

#### Adoption

Five state-based Cancer Councils were approached to take part in the study; four agreed and one declined due to resource limitations.

#### Reach

Participant flow through the study is detailed in Fig. 1. In total, 1183 cancer survivors were referred into HLaC (260 to 372 in each Cancer Council) with 886 (90.4% of those screened) found eligible to participate. The predominant referral pathway into the program was directly through the Cancer Councils (callers to their telephone information and support line, website visitors and/or users of other Cancer Council support services; Additional file 3). Ultimately, 786 eligible cancer survivors participated, an uptake of 88.7% overall (84.6 to 96.6% in each Cancer Council). Uptake did not differ significantly between referral sources or participant characteristics: age; sex; cancer type; and, time since diagnosis (Additional file 3).

Participant characteristics are shown in Table 1, overall and by each state Cancer Council. Overall, participants in the HLaC program (*n* = 786) were mostly women (88.0%), mostly Caucasian (89.7%), had an average (mean ± SD) age of 57.5 ± 11.4 years, BMI of 28.8 ± 6.5 kg/m^2^, were on average 1.9 ± 3.0 years since diagnosis, and many (44.3%) lived in areas with postcodes ranked in the highest 30% for their state regarding socioeconomic position. There were some apparent differences (≥10% or *p* < 0.05) between the four Cancer Councils in the sample of participants recruited. Some variation was seen in ethnicity (4.7 to 14.5% minority), degree of comorbidity (17.0 to 25.6% had ≥4 comorbidities), education (32.9 to 50.8% had a university education), employment (8.3 to 20.0% unable to work), geography (67.1 to 80.8% living in major cities), socioeconomic position (38.0 to 51.8% were in the middle 40% of postcodes for their state), cancer type (56.7 to 67.9% had breast cancer), receipt of hormone therapy (36.5 to 50.0%), mean age at baseline (55.3 to 59.5 years), and mean BMI (27.7 to 30.1 kg/m^2^). None of these differences were significant at *p* < 0.001. At baseline, many participants already had BMI < 25 kg/m^2^ (67.4%), many met recommendations of at least two daily fruit serves (55.1%) and 150 min/week moderate-vigorous physical activity (49.9%), while only a small minority had a low-risk waist circumference (14.5%; < 80 cm in women, < 94 cm in men) and few consumed at least 5 daily vegetable serves (16.5%) (Additional file 4).

#### Implementation

Study retention was 63.4%, with 498 participants completing the post-program evaluation and 288 dropping out. Program completion was 60.6% overall, with 476 participants completing ≥4 intervention calls and post-program evaluation and varied across the Cancer Councils (66.5% [A], 67.1% [B], 35.0% [C], and 75.6% [D], *p* < 0.001). The remaining 310 participants (39.4%) were classified as withdrawn, predominantly because they were uncontactable (30.3%), not interested (24.2%), too busy (22.9%), or for personal health reasons usually involving their cancer (18.7%), with a small number withdrawing for other reasons including family illness (3.9%). Differences between those who completed the program or withdrew were non-significant (*p* ≥ 0.001) and also mostly small (Additional file 5), with some minor tendencies for program completers (relative to their counterparts) to be older, from an English speaking background, have breast as opposed to other forms of cancer, have higher baseline fruit and vegetable intakes, and better mental quality of life. Program completers received a median of 11 calls (from 4 to 17) compared with 3 (0 to 13) calls among withdrawals. Mean (±SD) intervention call duration was 30.6 ± 10.6 min across the 4687 delivered calls whose duration was recorded. No serious adverse events related to the intervention were reported.

#### Costs

Program delivery costs were estimated at AU$504,980 (US$349,709) for the 1183 referred cancer survivors, equating to a mean cost of AU$427 (US$296) per referred cancer survivor (Additional file 1). The mean cost was AU$85 (US$59) per ineligible cancer survivor or whose eligibility we could not determine (*n* = 297), AU$388 (US$269) per partial program completer / did not commence the program (*n* = 410), and AU$673 (US$466) per program completer (*n* = 476).

### Maintenance

At the time of writing, each of the four HLaC participating Cancer Councils were considering HLaC results and delivery costs to inform decisions about whether or how they might continue to offer the program. During the study, the majority of program delivery was funded by the study grant. However, following the study, delivery costs would be fully borne by each Cancer Council. One Cancer Council was in the process of adapting the program for web-based delivery and two were going to continue to offer it at a reduced scale and as a means of promoting maintenance among cancer survivors completing their existing exercise classes.

### Secondary outcomes

#### Effectiveness

All of the patient-reported outcomes improved significantly over time (Table 2) and did not significantly differ between the Cancer Councils. Overall, average changes were − 2.24 kg body weight (95% CI: − 2.61, − 1.88), equivalent to − 0.80 kg/m^2^ BMI (95% CI: − 0.93, − 0.67). These corresponded with substantial improvements in self-reported MVPA (148 min/week, 95% CI: 125, 171), reduced sitting time (− 1.19 h/day, 95% CI: − 1.42, − 0.96), and small (0.2 to < 0.5 SD) to moderate (0.5 to < 0.8 SD) improvements in dietary outcomes: an increase of 0.99 vegetable serves/day (95% CI: 0.83, 1.16); 0.28 fruit serves/day (95% CI: 0.29, 0.36); 0.32 units on the dietary fat index (95% CI: 0.29, 0.36); and, 0.24 units on the dietary fibre index (95% CI: 0.19, 0.28). There were also sizeable improvements (> 0.5 SD) in physical quality of life (6.10 units, 95% CI: 5.21, 7.00), symptom severity (− 1.00 units, 95% CI: − 1.12, − 0.87) and symptom interference (− 1.36 units, 95% CI: − 1.53, − 1.18). There were also small improvements (0.2 to < 0.5 SD) in psychosocial outcomes: mental quality of life (2.66 units, 95% CI: 1.80, 3.51); fear of cancer recurrence (− 3.36 units, 95% CI:-4.07, − 2.65); distress level (− 0.71, 95% CI: − 0.94, − 0.48); and, distress impact (− 0.68 units, 95% CI: − 0.90, − 0.47). The degree to which these changes in mean outcomes corresponded with increases in the proportion of participants adhering to national recommendations can be seen in Additional file 4.

**Table 2:** Baseline to post-program changes in patient reported outcomes in all Healthy Living after Cancer participants

Outcome	M ± SD at baseline	n		Pooled mean change (95%CI) ^**a**^	p	p for interaction (time x cancer council)
		Baseline	Post			
Weight, kg	78.9 ± 18.8	786	494	−2.24 (−2.61, −1.88)	< 0.001	*0.057*
Body Mass Index, kg/m^2^	28.8 ± 6.5	786	494	−0.80 (−0.93, −0.67)	< 0.001	*0.081*
Waist circumference, cm	97.6 ± 15.3	781	477	−4.42 (−5.08, −3.77)	< 0.001	*0.063*
MVPA, min/week	207.4 ± 210.2	786	498	147.64 (124.25, 171.03)	< 0.001	0.334
Sitting on weekdays, h/day	6.5 ± 3	782	498	−1.19 (− 1.42, −0.96)	< 0.001	0.755
Vegetables, serves/day	3.0 ± 1.8	786	498	0.99 (0.83, 1.16)	< 0.001	0.111
Fruit, serves/day	1.8 ± 1.1	785	498	0.28 (0.19, 0.36)	< 0.001	*0.074*
Fat Index, 1–5	3.1 ± 0.5	771	489	0.32 (0.29, 0.36)	< 0.001	0.255
Fibre Index, 1–5	2.8 ± 0.5	753	461	0.24 (0.19, 0.28)	< 0.001	0.290
Physical Quality of life, 0–100	39.7 ± 10.2	786	500	6.10 (5.21, 7.00)	< 0.001	0.277
Mental Quality of life, 0–100	47.2 ± 10.7	786	500	2.66 (1.80, 3.51)	< 0.001	0.184
Symptom Severity, 0–10	4.1 ± 1.8	786	499	−1.00 (−1.12, −0.87)	< 0.001	0.655
Symptom Interference, 0–10	3.9 ± 2.4	785	499	−1.36 (− 1.53, − 1.18)	< 0.001	0.687
Fear of cancer recurrence, 0–40	16.5 ± 10.4	786	499	−3.36 (−4.07, −2.65)	< 0.001	0.503
Distress Level, 0–10	2.9 ± 2.7	784	497	−0.71 (− 0.94, − 0.48)	< 0.001	0.891
Distress Impact, 0–10	2.2 ± 2.8	784	497	−0.68 (− 0.90, − 0.47)	< 0.001	0.402

*MVPA* moderate-vigorous physical activity, *CI* confidence interval

^a^ Pooled mean change estimated by comparison of marginal means for the effect of time (baseline / post program), estimated balanced across strata (cancer council: A, B, C, D) from model that includes effects of time, cancer council, and their two-way interaction

Stratified results are shown in Additional file 6. As with the pooled effects, the direction of the changes consistently favoured improvement, though not always statistically significant or necessarily of the same magnitude. Importantly, none of the Cancer Councils achieved stronger or weaker results across all or most outcomes.

Additional file 7 shows the pooled effects as estimated from all participants (multiple imputation analysis), in evaluable cases with adjustment for predictors of dropout, and in those who adhered to the program protocol (i.e., program completers). Conclusions were mostly robust to missing data handling, with all of the outcomes improving significantly and to a similar extent: most were identical to within ±10% in multiply imputed and dropout-adjusted models relative to what was seen in the main analyses. Improvements in fruit intake were ≈14% smaller (0.04 serves) in both sensitivity analyses; improvements in distress were ≈13% lower with adjustment for predictors of dropout (0.09 units), and improvement in mental quality of life were ≈12% lower with adjustment for predictors of dropout (0.30 units). The per protocol analysis showed similar results in those who adhered to the protocol (i.e., were program completers) to those seen in all evaluated participants. All outcomes improved significantly, to a similar degree as in the main evaluation (most were identical to within ±10%, with the largest difference in results being for distress impact (≈19% smaller improvement in program completers than overall). Per-protocol results within each Cancer Council are shown in Additional file 8. As with the main evaluation, these showed all outcomes tended towards improvement in every Cancer Council (not always statistically significant), and with some potentially sizable (but not statistically significant) differences between the Cancer Councils.

#### Participant satisfaction

**Fig. 2:**
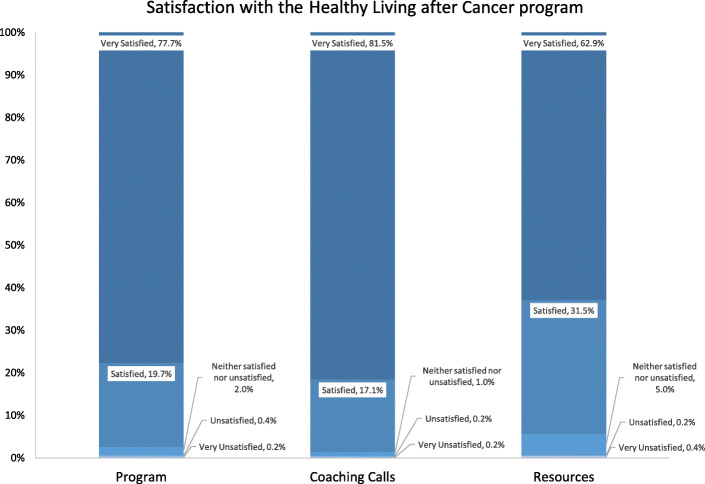
Participant satisfaction with the Healty Living after Cancer program

Figure 2 shows participant satisfaction with the program, coaching calls and resources. Overall, satisfaction was very high with 77.7, 81.5 and 62.9% reporting the highest satisfaction rating “very satisfied” for these aspects of the HLaC Program, respectively.

## Discussion

The Healthy Living after Cancer program was taken up by four of five state-based Australian Cancer Councils, delivered to 786 (≈89%) eligible referred cancer survivors, most of whom (80%) received the minimum desired health coaching and many of whom (60%) completed the program. While some of the primary outcomes (especially completion rates) varied between the Cancer Councils, effectiveness of the program was demonstrated by all participating Cancer Councils. The program was significantly effective for all of the participant-reported outcomes. Improvements in body anthropometry (e.g., ≈2 kg weight loss) occurred alongside sizeable increases in physical activity and modest improvements in dietary indicators, with corresponding sizeable improvements in physical quality of life and symptoms, and smaller improvements in mental quality of life and other psychosocial outcomes.

Direct comparators for HLaC primary outcomes of *reach and implementation* are few, as there are no published reports of health behaviour interventions for cancer survivors delivered via distance modalities and at scale by health professional staff in a health service setting. Indeed, retention rates in implementation or real-world contexts are typically lower than that observed in controlled trials. The most comparable program is the Australian Get Healthy Information and Coaching Service [49, 50]. Like HLaC, the Get Healthy Service is a six-month telephone health coaching program targeting physical activity, healthy eating and healthy weight. However, it is delivered by a state health department and targets the general adult population, primarily through media-based promotion.

The Get Healthy Service reported a program completion rate of approximately 25%, substantially lower than that achieved in HLaC (60%). This is likely due to the significant challenge of attracting and retaining program participants made aware of the program primarily through advertising, rather than targeted accrual through personal invitation. HLaC participants were mostly those who had contacted the Cancer Council in their state for information about cancer and its effects and as such may have had a higher level of motivation for program completion. Higher completion rates (44%) were also seen in a six-month telephone health coaching program delivered by an Australian Division of General Practice to adults with (non-cancer) chronic conditions and based on general practitioner referral [51], suggesting the importance of more targeted forms of referral. It may also be the case that those with a diagnosis of cancer are more motivated to complete health behaviour intervention programs than the general adult population or adults with other (non-cancer) chronic conditions. This has been reported particularly among women with breast cancer, with study retention rates typically 80–90% [25, 52], noting that the majority of HLaC participants were women with breast cancer.

In terms of secondary *effectiveness* outcomes, mean weight loss observed in HLaC is less than what was observed in our precursor randomised controlled effectiveness trials in women with breast cancer [53] and other similar telephone-delivered breast cancer weight loss trials [54]. However, this is consistent with the attenuation of intervention effect sizes when intervention evaluation occurs in efficacy, effectiveness and dissemination contexts [55]. Evidence suggests that weight losses of 7–10% of body weight are likely needed to reduce risk of co-morbidities and mortality [56], although benefit may be seen with weight loss as little as 3% [57]. Thus the magnitude of weight loss observed in HLaC is likely to impart some health benefit, particularly on a population level, given the scalable nature of the intervention, and the detrimental effects of this weight gain on both cancer and general health outcomes [58].

HLaC mean *costs* are considered to be relatively low when taking into account the improvements observed in weight management, physical activity and diet. They are in line with the limited reporting of intervention delivery costs in cancer survivor populations [28, 59] and similar to costs reported in the Get Healthy Service evaluation described below [49].

In comparison to HLaC *effectiveness* outcomes, weight, waist circumference and dietary outcomes (the only ones directly comparable with Get Healthy Service reporting and based on participant self-report), Get Healthy Service outcomes are marginally superior for weight and waist circumference (− 3.8 kg and − 5.1 cm, respectively), but similar for vegetable and fruit serves (+ 1.2 and + 0.4 serves, respectively; GHS evaluation report) [50]. Greater decreases in weight and waist circumference may be due to the higher levels of the Get Healthy Service sample at baseline and perhaps also owing to the selected sample of program completers.

Lawler and colleagues, in a pre-post pilot study, offered the Get Healthy Service to 53 women with stage I-III breast cancer following treatment and on referral by cancer nurses in an Australian breast cancer clinic [60]. Here, among program completers, results for program completion and self-reported weight were similar to HLaC (62% and − 2.4 kg, respectively), increases in minutes per week of MVPA were less (+ 55 min) and no increase in serves of vegetables or fruit were observed. The increase in weekly MVPA observed in HLaC (+ 148 min) is particularly notable, as it is consistent with a magnitude of benefit shown in epidemiologic studies to confer a significant reduction in cancer mortality [61].

The primary strength of the study was the partnership context in which it was conducted. This included working closely with the Cancer Councils to integrate the intervention into their Cancer Information and Support Service to achieve delivery at scale. It also afforded opportunity to build capacity amongst Cancer Council staff in lifestyle intervention implementation and program evaluation. Making the program widely available across the diverse population of cancer survivors was also a strength. The generalisability of results is limited by the largely female, Caucasian and breast cancer survivor sample, which does not represent the broader population of Australian cancer survivors [2]. There are also inherent limitations in the use of a single group, pre-post study design, along with the use of self-report outcome measures. The collection of data by Cancer Council staff, some of whom had a role in program delivery, could have been a source of bias, but this risk is mitigated by the robustness of findings across strata. The evaluation of effectiveness was adequately powered overall but lacked precision around estimated effectiveness within each Cancer Council, as well as the comparison between the Cancer Councils.

## Conclusions

This is the first study that reports on the effectiveness and feasibility of a scaled up and national implementation of an evidence-based, telephone-delivered, lifestyle program for cancer survivors implemented in conjunction with a peak cancer control partner. It was designed in response to calls for the conduct of practice-based dissemination research that accelerates the transfer of evidence into cancer survivorship care [62–64]. Based on our RE-AIM findings, the intervention is considered feasible to deliver at scale, with improvements in anthropometric, behavioural and psychosocial participant-reported outcomes of a magnitude likely to reduce cancer morbidity and mortality in the growing number of cancer survivors. While the costs to deliver the program were relatively low, particularly in light of participant benefits, all Cancer Councils indicated that the resource implications of sustained program delivery remained a barrier. At the time of writing, two Cancer Councils were delivering the program on a smaller scale and one was in the process of adapting it for online delivery. Advocacy efforts targeting funding from state and national government health and cancer agencies, based on the strong evidence of impact demonstrated in this dissemination study, will likely be required to support continued program delivery.

## Supplementary information

**Additional file 1 **Program costs. **Table 1.** Estimated delivery costs of the Healthy Living after Cancer project for 1183 participants across four participating sites over 48 months.

**Additional file 2 : Table 2.** Variables included in multiple imputation analyses as predictors of missingness or auxiliary variables.

**Additional file 3 : Table 3.** Comparison of eligible participants versus eligible non-participants.

**Additional file 4 : Table 4.** Percentage of participants meeting recommendations before and after Healthy Living after Cancer.

**Additional file 5 : Table 5.** Comparison of those who met Healthy Living after Cancer program completion criteria (*n* = 476) with those who withdrew (*n* = 310).

**Additional file 6 : Table 6.** Patient-reported outcomes in Healthy Living after Cancer participants by Cancer Council (evaluable case analysis).

**Additional file 7 : Table 7.** Baseline (pre) to post-program changes in patient reported outcomes in Healthy Living after Cancer participants (sensitivity analyses).

**Additional file 8 : Table 8.** Patient-reported outcomes in Healthy Living after Cancer program completers by Cancer Council (per-protocol analysis, evaluable cases).

## Data Availability

The datasets used and/or analysed during the current study are available from the corresponding author on reasonable request.

## Abbreviations

HLaC: Healthy living after cancer
RE-AIM: Reach, effectiveness, adoption, implementation and maintenance framework
BMI: Body mass index
MVPA: Moderate-to-vigorous physical activity
