# Supplementary material for: Translating research into practice: outcomes from the Healthy Living after Cancer partnership project
Source: BMC Cancer. 2020 Oct 6;20:963. doi: 10.1186/s12885-020-07454-4 (PMC7539431; doi:10.1186/s12885-020-07454-4)
Supplement: Supplementary file 1 — Additional file 1 Program costs. Table 1. Estimated delivery costs of the Healthy Living after Cancer project for 1183 participants across four participating sites over 48 months. [file 12885_2020_7454_MOESM1_ESM.docx]

**Additional File 1 – Program costs**

We estimated the costs associated with implementing the Healthy Living after Cancer (HLaC) program across the four participating sites for the trial period from an organisational perspective. Project records and participating Cancer Councils provided the monetary values (2015-2017) and quantities for service delivery resources required to deliver the HLaC program: salaries, participant workbooks, marketing resources, training and telephone expenses. Commercial prices were used for the production of training manuals, envelopes and postage. Staff time to complete referral, screening, consent, assessment and intervention calls and associated administration were recorded for each participant by the Cancer Councils or using periodic audits. Costs to conduct brief pre- and post-assessment calls were estimated at 50% of the call and admin staff time to reflect a possible sustainable approach to monitoring participant outcomes. Costs associated with research, recruiting staff, office and telephone rental, modifying databases, wrap up calls and refresher group-based training were excluded from analyses.

Costs included advertising materials, staff training, personnel costs, participant materials and telephone charges. Variable costs varied according to the number of staff (e.g. training manuals, staff training), the number of participants (e.g. staff call and administration time to deliver the program, telephone expenses, participant workbooks and office consumables), or were required on an on-going basis (e.g. staff time for debriefing and refining referral pathways, and marketing materials).

Of the 1,183 referred participants, a total of 297 participants were ineligible, 410 participants partially completed and/or did not commence the program, and 476 participants completed the program. Service delivery costs of implementing the HLaC program at the four participating Cancer Councils over the 48-month trial period were generated and are presented in Additional Table 1.

Overall, the cost to implement the HLaC program was AU$504,980 (US$349,709) across the trial sites. This equated to AU$427 (US$296) per referred cancer survivor (n=1,183). For the 297 ineligible cancer survivors, the mean cost was AU$85 (US$59) per cancer survivor. For the 410 partial program completers / did not commence the program, the mean cost was AU$388 (US$269) per cancer survivor. For the 476 program completers, the mean cost was AU$673 (US$466) per cancer survivor.

Staff costs accounted for the highest proportion of costs (88%) which included staff time to deliver the program (calls and administration), attend debriefing sessions and refine referral pathways. The majority (74%) of the staff time to deliver the program was spent on the intervention phase of the program. The remaining staff call and administration time was spent on conducting referral, screening and consent calls (17%) and brief pre- and post-assessments (10%). The administration time may be overestimated as no formal definition for the administrative tasks directly associated with the delivery of the HLaC program was provided.

Additional Table 1: Estimated delivery costs of the Healthy Living after Cancer project for 1,183 participants across four participating sites over 48 months

|  |  | **Program costs (AU$)** | | | **Total** |
| --- | --- | --- | --- | --- | --- |
| **Resources** | **Details of quantities and values** | **Ineligible** | **Eligible** | |  |
|  |  | **Ineligible** | **Partial completers & did not commence** | **Completers** |  |
|  |  | (n=297) | (n=410) | (n=476) | (n=1,183) |
| *Marketing materials* |  |  |  |  |  |
| Brochures | 1,341 brochures/month @ $0.08 each (40 months)^1^ | $1,037 | $1,431 | $1,662 | $4,130 |
| Posters | 5 posters/month @ $1.49 each (40 months) ^1^ | $75 | $103 | $120 | $298 |
| *Staff training* |  |  |  |  |  |
| Staff training^2^ | 2-day group-based workshop & self-directed training^1^ | $6,822 | $9,417 | $10,933 | $27,171 |
| Training manuals | 21 staff x 1 training manual per staff @ $25.02 each^1^ | $132 | $182 | $211 | $525 |
| *Personnel costs* |  |  |  |  |  |
| Program (Call time)^3^ | Staff @ $50/hour (incl. on-costs) x 4,757 hours^4^ | $6,862 | $60,252 | $170,730 | $237,844 |
| Program (Admin time)^5^ | Staff @ $50/hour (incl. on-costs) x 1,900 hours^6^ | $4,080 | $30,327 | $60,607 | $95,015 |
| Personnel – Debriefing^7^ | Staff @ $53/hour x 34 hrs/month (48 months) | $0 | $40,675 | $47,223 | $87,899 |
| Referral pathways | Staff @ $47/hr x 3hrs/month/site x 4 sites (40 months) ^1^ | $5,662 | $7,816 | $9,074 | $22,552 |
| *Participant materials* |  |  |  |  |  |
| Workbook^8^ | $8.11 each (1 workbook per pre-assessment) | $41 | $2,605 | $3,862 | $6,507 |
| Envelopes^8^ | $0.15 each (C4 envelope for workbook) | $1 | $48 | $71 | $120 |
| Postage^8^ | $5 each (postage for workbook) | $25 | $1,605 | $2,380 | $4,010 |
| *Telephone charges* |  |  |  |  |  |
| Telephone charges | 14,271 calls (50% local & 50% mobile calls) | $550 | $4,815 | $13,543 | $18,909 |
| *Total cost of HLaC program (AU$)* | | *AU $25,286* | *AU $159,277* | *AU $320,417* | *AU $504,980* |
| *Total cost of HLaC program (US$)* | | *US $17,511* | *US $110,303* | *US $221,896* | *US $349,709* |
| *Cost per participant (AU$)* | | *AU $85* | *AU $388* | *AU $673* | *AU $427* |
| *Cost per participant (US$)* | | *US $59* | *US $269* | *US $466* | *US $296* |

Resources were valued in Australian 2015-17 Australian dollars and converted to United States dollars (1 USD = 1.444 AUD) adjusted to 2017 prices using the OECD purchasing power parities (PPP) converter: https://data.oecd.org/conversion/purchasing-power-parities-ppp.htm#indicator-chart.

^1^ The total item cost is apportioned across the 3 groups based on participants per group (i.e. ineligible, n=297; partial completers/did not commence, n=410; Completers, n=476).

^2^ Training workshop costs included staff time, travel, accommodation, catering and incidentals (3 facilitators; 8 staff). Self-directed training costs were estimated as staff time for two days to review the training videos and materials for staff that did not attend the formal training workshop (11 staff).

^3^ Includes referral, screening, consent, intervention, brief pre- and post-assessments (50% duration) call time for delivered and non-delivered sessions.

^4^ Based on 137 hours (ineligible), 1,205 hours (partial completers/did not commence), 3,415 hours (completers) and 4,757 hours (total).

^5^ Includes referral, screening, consent, intervention, brief pre- and post-assessments (50% duration) call time for delivered and non-delivered sessions.

^6^ Based on 82 hours (ineligible), 607 hours (partial completers/did not commence), 1,212 hours (completers) and 1,900 hours (total).

^7^ Includes debriefing meetings, administration, call monitoring and mentoring.

^8^ Participants that completed the pre-program assessment were posted a participant workbook (ineligible, n=5; partial completers/did not commence, n=321; completers, n=476).
