# Supplementary material for: Translating research into practice: outcomes from the Healthy Living after Cancer partnership project
Source: BMC Cancer. 2020 Oct 6;20:963. doi: 10.1186/s12885-020-07454-4 (PMC7539431; doi:10.1186/s12885-020-07454-4)
Supplement: Supplementary file 2 — Additional file 2 : Table 2. Variables included in multiple imputation analyses as predictors of missingness or auxiliary variables. [file 12885_2020_7454_MOESM2_ESM.docx]

Additional Table 2: Variables included in multiple imputation analyses as predictors of missingness or auxiliary variables

| Characteristic | Predictor of missingness ^a^ | Outcome ^b^ | | | | | | | | | | | | | | | |
| --- | --- | --- | --- | --- | --- | --- | --- | --- | --- | --- | --- | --- | --- | --- | --- | --- | --- |
|  |  | A1 | A2 | A3 | PA1 | PA2 | D1 | D2 | D3 | D4 | Q1 | Q2 | S1 | S2 | P1 | P2 | P3 |
| Cancer Council | All | A | A | A | A |  |  |  | A |  |  | A | A |  |  | A |  |
| Age, years | All |  |  |  |  |  |  | A |  |  |  |  |  |  |  |  |  |
| Sex *(male / female)* |  | A |  |  | A |  | A |  |  | A |  | A |  |  |  |  |  |
| Anthropometry: Weight (W), BMI or waist circumference (WC) | F, WC |  |  | W  WC | BMI | BMI |  | BMI | BMI | BMI WC |  |  |  |  |  |  |  |
| Referral source *(Cancer Council / other health / media / word of mouth or other)* | FCR, WC | A | A |  | A |  |  |  |  |  |  |  |  |  |  |  |  |
| Major City *(yes / no)* | All |  |  |  |  |  |  | A |  | A | A |  |  | A |  |  |  |
| IRSAD *(bottom 30% / middle 40% / top 30%) ^c^* | All | A | A | A |  |  | A |  |  |  | A | A |  |  |  |  |  |
| Marital status *(married or living together / widowed, separated or divorced / never married)* | All |  |  |  |  |  |  | A | A | A |  |  |  | A | A |  | A |
| Ethnicity *(Caucasian / other)* | All |  |  |  |  |  |  |  |  |  |  |  |  |  | A | A | A |
| Non-English speaking background *(yes / no)* | All |  |  |  | A |  |  |  |  |  |  |  |  |  |  |  |  |
| Education *(< high school / high school / trade or technical / ≥ university)* | DO, FCR |  |  |  | A |  |  |  | A |  | A |  |  |  |  |  |  |
| Employment *(full time / part time or casual / home duties / retired / unable to work / other not working / other working [self-employed])* |  |  |  |  |  | A | A |  | A | A | A |  |  | A |  |  |  |
| Smoking *(never / previous / current)* | All |  |  |  |  |  | A |  |  |  |  |  |  |  | A |  |  |
| Alcohol, standard drinks/week |  |  |  |  | A |  | A |  |  |  |  |  |  |  |  |  |  |
| MVPA, min/week | FCR, WC |  |  |  | A |  | A | A |  | A |  |  |  | A |  |  |  |
| Sitting, h/day |  |  |  |  | A | A |  |  |  |  |  |  |  |  |  |  |  |
| Fruit intake, serves/day | All |  |  |  | A | A |  | A |  |  |  | A |  |  |  |  |  |
| Vegetable intake, serves/day | All |  |  |  |  |  | A |  |  |  |  |  |  |  |  |  |  |
| Fat Index (1-5) |  | A | A | A | A |  |  | A | A | A | A |  | A | A |  | A | A |
| Fibre Index (1-5) | FCR |  |  |  |  |  |  |  |  | A |  |  |  |  |  |  |  |
| Physical Quality of Life (0-100) |  |  |  |  |  |  |  |  |  |  | A | A |  | A |  |  |  |
| Mental Quality of Life (0-100) | All |  | A |  |  |  |  |  | A |  |  | A |  |  |  |  | A |
| Symptom Severity (0-10) | All | A | A |  | A |  |  |  |  |  | A |  |  | A | A |  | A |
| Symptom Interference (0-10) | All |  |  | A |  |  |  | A |  |  |  | A | A | A |  |  | A |
| Fear of Cancer Recurrence (0-40) | All |  |  | A |  |  |  |  |  |  |  |  | A |  | A |  | A |
| Distress Level (0-10) | All |  |  |  |  |  | A |  | A | A |  |  |  | A |  |  | A |
| Distress Impact (0-10) | All |  |  |  | A | A | A |  | A | A | A | A | A | A | A |  |  |
| Cancer type *(breast / colorectal or bowel / prostate / other)* | All | A | A |  |  |  |  | A |  | A |  |  | A | A |  |  | A |
| Years since diagnosis | DO, WC | A | A |  |  |  |  |  | A | A |  |  |  | A |  |  | A |
| Chemotherapy *(yes / no)* | F, FCR, WC | A | A |  |  | A | A |  | A |  |  |  |  | A |  |  |  |
| Radiotherapy *(yes / no)* | F |  |  |  |  |  |  |  |  |  |  |  | A | A |  |  | A |
| trastuzumab *(yes / no)* | DO, F, FCR |  |  |  | A |  | A |  | A |  |  |  |  |  |  |  |  |
| Hormone therapy *(yes / no)* |  | A | A |  | A |  |  |  |  |  | A |  |  |  |  |  |  |
| Number of comorbidities | All |  |  |  |  |  |  | A |  |  | A |  | A | A | A |  |  |

MVPA = moderate-vigorous physical activity; Variables included in each multiple imputation model are shaded in grey, with multiple imputation performed using Fully Conditional Specification with predictive mean matching in SPSS v24 with m=50 imputations

^a^ Association at *p* < 0.2 with missing data: Dropout (DO), Fibre Index Score (F), Missing 1+ Fear of Cancer Recurrence items (FCR), Missing Waist circumference (WC) or all of these (All).

^b^ Outcomes were A1 = weight, A2 = BMI [Body Mass Index] A3 = Waist circumference, PA1 = Moderate-Vigorous Physical Activity, PA2 = Sitting, D1 = Vegetable intake, D2 = Fruit Intake, D3 = Fat Index, D4 = Fibre index, Q1 = Physical Quality of Life, Q2 = Mental Quality of Life, S1 = Symptom Severity, S2 = Symptom Interference, P1 = Fear of Cancer Recurrence, P2 = Distress Level, P3 = Distress Impact

^c^ Postcode Index of Relative Socioeconomic Advantage and Disadvantage (IRSAD) State Based Percentiles
