# Supplementary material for: Translating research into practice: outcomes from the Healthy Living after Cancer partnership project
Source: BMC Cancer. 2020 Oct 6;20:963. doi: 10.1186/s12885-020-07454-4 (PMC7539431; doi:10.1186/s12885-020-07454-4)
Supplement: Supplementary file 3 — Additional file 3 : Table 3. Comparison of eligible participants versus eligible non-participants. [file 12885_2020_7454_MOESM3_ESM.docx]

Additional Table 3: Comparison of eligible participants versus eligible non-participants

|  | **n** | **Participant** | **n** | **Refused (eligible)** | **p** |
| --- | --- | --- | --- | --- | --- |
| Age (at referral) ^a^ | 786 | 55.02 ± 11.33 | 94 | 52.64 ± 12.64 | 0.058 |
|  |  |  |  |  |  |
| Time since diagnosis (at referral) | 786 | 1.84 ± 3.03 | 84 | 2.08 ± 3.29 | 0.490 |
|  |  |  |  |  |  |
| Referral Source |  |  |  |  | 0.691 |
| 13 11 20 personnel | 280 | 35.6% | 40 | 40.8% |  |
| Cancer Council website | 77 | 9.8% | 11 | 11.2% |  |
| Other Cancer Council service/program | 155 | 19.7% | 19 | 19.4% |  |
| Health professional | 139 | 17.7% | 19 | 19.4% |  |
| Other cancer services/program | 27 | 3.4% | 1 | 1.0% |  |
| Media | 97 | 12.3% | 7 | 7.1% |  |
| Friends/family/word of Mouth | 10 | 1.3% | 1 | 1.0% |  |
| Other source | 1 | 0.1% | 0 | 0.0% |  |
| Sex |  |  |  |  | 0.180 |
| Male | 94 | 12.0% | 7 | 7.1% |  |
| Female | 692 | 88.0% | 90 | 92.9% |  |
| Cancer type |  |  |  |  | 0.131 |
| Breast | 484 | 61.6% | 61 | 68.5% |  |
| Colorectal/bowel | 71 | 9.0% | 8 | 9.0% |  |
| Lymphoma | 66 | 8.4% | 9 | 10.1% |  |
| Prostate | 30 | 3.8% | 3 | 3.4% |  |
| Other | 135 | 17.2% | 8 | 9.0% |  |
| Cancer Council ^b^ |  |  |  |  | <0.001 |
| A | 248 | 31.6% | 45 | 45.0% |  |
| B | 170 | 21.6% | 6 | 6.0% |  |
| C | 200 | 25.4% | 21 | 21.0% |  |
| D | 168 | 21.4% | 28 | 28.0% |  |

^a^ Age at referral, truncated at 80 years, using midpoint of 10-year age categories when reported as a range only.

^b^ The corresponding % participation among eligible referred survivors within Cancer Councils A–D are 84.6%, 96.6%, 90.5%, and 85.7%, respectively
