# Supplementary material for: Translating research into practice: outcomes from the Healthy Living after Cancer partnership project
Source: BMC Cancer. 2020 Oct 6;20:963. doi: 10.1186/s12885-020-07454-4 (PMC7539431; doi:10.1186/s12885-020-07454-4)
Supplement: Supplementary file 4 — Additional file 4 : Table 4. Percentage of participants meeting recommendations before and after Healthy Living after Cancer. [file 12885_2020_7454_MOESM4_ESM.docx]

Additional Table 4: Percentage of participants meeting recommendations before and after Healthy Living after Cancer

|  | **Pre intervention (all)** | | **Pre intervention ^a^** | | **Post intervention ^a^** | | |
| --- | --- | --- | --- | --- | --- | --- | --- |
|  | **n** | **Summary** | **n** | **Summary** |  | **Summary** |  |
| Fruit ≥ 2 serves/day | 785 | 433 (55.1%) | 497 | 290 (58.4%) | 497 | 372 (74.8%) |  |
| Vegetables ≥ 5 serves/day | 786 | 130 (16.5%) | 498 | 90 (18.1%) | 498 | 190 (38.2%) |  |
| MVPA ≥ 150 min/week | 786 | 392 (49.9%) | 498 | 249 (50.0%) | 498 | 394(79.1%) |  |
| BMI <25 kg/m^2^ | 786 | 530 (67.4%) | 494 | 328 (66.4%) | 494 | 313 (63.4%) |  |
| Waist circumference ^b^ | 781 | 114 (14.5%) | 476 | 64 (13.4%) | 476 | 109 (22.9%) |  |

MVPA = Moderate-vigorous physical activity; BMI = Body Mass Index

^a^ Those who provided data at both pre and post intervention assessments; ^b^ <80 cm (women) < 94 cm (men)
