# Supplementary material for: Translating research into practice: outcomes from the Healthy Living after Cancer partnership project
Source: BMC Cancer. 2020 Oct 6;20:963. doi: 10.1186/s12885-020-07454-4 (PMC7539431; doi:10.1186/s12885-020-07454-4)
Supplement: Supplementary file 5 — Additional file 5 : Table 5. Comparison of those who met Healthy Living after Cancer program completion criteria (n = 476) with those who withdrew (n = 310). [file 12885_2020_7454_MOESM5_ESM.docx]

Additional Table 5: Comparison of those who met Healthy Living after Cancer program completion criteria (n=476) with those who withdrew (n=310)

| **Characteristic** | **Completed program ^a^** | | **Withdrew** | | ***p*** |
| --- | --- | --- | --- | --- | --- |
|  | n | M ± SD or n (%) | n | M ± SD or n (%) |  |
| Age, years | 475 | 58.54 ± 10.91 | 310 | 55.95 ± 12.05 | 0.002 |
| BMI, kg/m^2^ | 476 | 28.6 ± 6.16 | 310 | 29.13 ± 7.01 | 0.269 |
| Physical Quality of Life (0-100) | 476 | 39.88 ± 10.21 | 310 | 39.48 ± 10.32 | 0.595 |
| Mental Quality of Life (0-100) | 476 | 48.27 ± 10.37 | 310 | 45.60 ± 11.03 | 0.001 |
| Female, n (%) | 476 | 425 (89.3%) | 310 | 267 (83.1%) | 0.216 |
| Caucasian, n (%) | 475 | 272 (88.0%) | 309 | 433 (91.2%) | 0.182 |
| Non-English speaking background |  | 260 (83.9%) |  | 425 (89.5%) | 0.028 |
| Highest education completed | 476 |  | 310 |  | 0.222 |
| < High School |  | 54 (17.4%) |  | 61 (12.8%) |  |
| High School |  | 28 (9.0%) |  | 43 (9.0%) |  |
| Technical / Trade |  | 95 (30.6%) |  | 172 (36.1%) |  |
| University or postgraduate |  | 133 (42.9%) |  | 200 (42.0%) |  |
| Employed | 476 | 213 (59.0%) | 310 | 272 (57.1%) | 0.559 |
| Married / living together | 476 | 312 (65.5%) | 310 | 189 (61.0%) | 0.198 |
| IRSAD | 468 |  | 308 |  | 0.266 |
| Bottom 30% of state |  | 72 (15.4%) |  | 61 (19.8%) |  |
| Middle 30% of state |  | 182 (38.9%) |  | 117 (38%) |  |
| Top 30% of state |  | 214 (45.7%) |  | 130 (42.2%) |  |
| Reside in Major City | 470 | 243 (78.9%) | 308 | 351 (74.7%) | 0.266 |
| MVPA, min/week | 476 | 215 ± 220 | 310 | 196 ± 195 | 0.191 |
| Vegetables, serves/day | 476 | 3.15 ± 1.87 | 310 | 2.77 ± 1.58 | 0.004 |
| Fruit, serves/day | 475 | 1.83 ± 1.13 | 310 | 1.65 ± 1.03 | 0.031 |
| Alcohol (standard drinks/week) | 476 |  | 310 |  | 0.876 |
| 0 |  | 235 (49.4%) |  | 155 (50%) |  |
| >0 to <21 |  | 227 (47.7%) |  | 143 (46.1%) |  |
| 21 to <35 |  | 11 (2.3%) |  | 10 (3.2%) |  |
| ≥35 |  | 3 (0.6%) |  | 2 (0.6%) |  |
| Smoking status | 476 |  | 310 |  | 0.060 |
| Current |  | 16 (3.4%) |  | 18 (5.8%) |  |
| Previous |  | 171 (35.9%) |  | 127 (41.0%) |  |
| Never |  | 289 (60.7%) |  | 165 (53.2%) |  |
| Years since diagnosis | 476 | 2.01 ± 3.52 | 310 | 1.76 ± 2.02 | 0.206 |
| ≥4 Comorbidities, n (%) | 476 | 97 (20.4%) | 310 | 57 (18.4%) | 0.521 |
| Surgery, n (%) | 476 | 421 (88.4%) | 310 | 265 (85.5%) | 0.230 |
| Chemotherapy, n (%) | 476 | 312 (65.5%) | 310 | 219 (70.6%) | 0.139 |
| Radiotherapy, n (%) | 476 | 294 (61.8%) | 310 | 175 (56.5%) | 0.157 |
| Hormone therapy, n (%) | 476 | 210 (44.1%) | 310 | 133 (42.9%) | 0.769 |
| trastuzumab, n (%) | 476 | 51 (10.7%) | 310 | 24 (7.7%) | 0.174 |
| Cancer type | 476 |  | 310 |  | 0.055 |
| Breast |  | 307 (64.5%) |  | 177 (57.1%) |  |
| Colorectal / bowel |  | 36 (7.6%) |  | 35 (11.3%) |  |
| Lymphoma |  | 38 (8.0%) |  | 28 (9.0%) |  |
| Prostate |  | 22 (4.6%) |  | 8 (2.6%) |  |
| Other |  | 73 (15.3%) |  | 62 (20.0%) |  |
| Referral source | 476 |  | 310 |  | 0.296 |
| Cancer Council |  | 316 (66.4%) |  | 196 (63.2%) |  |
| Health professional / cancer service |  | 96 (20.2%) |  | 70 (22.6%) |  |
| Media |  | 55 (11.6%) |  | 42 (13.5%) |  |
| Word of mouth / other |  | 9 (1.9%) |  | 2 (0.6%) |  |

IRSAD = Index of Relative Socioeconomic Advantage and Disadvantage; MVPA = moderate-vigorous physical activity

^a^ ≥ 4 intervention calls and a post-program assessment
