# Supplementary material for: Translating research into practice: outcomes from the Healthy Living after Cancer partnership project
Source: BMC Cancer. 2020 Oct 6;20:963. doi: 10.1186/s12885-020-07454-4 (PMC7539431; doi:10.1186/s12885-020-07454-4)
Supplement: Supplementary file 6 — Additional file 6 : Table 6. Patient-reported outcomes in Healthy Living after Cancer participants by Cancer Council (evaluable case analysis). [file 12885_2020_7454_MOESM6_ESM.docx]

Additional Table 6: Patient-reported outcomes in Healthy Living after Cancer participants by Cancer Council (evaluable case analysis)

| Outcome | A | | B | | C | | D | |
| --- | --- | --- | --- | --- | --- | --- | --- | --- |
|  | n 1^a^ | Mean change (95% CI)^b^ | n 1^a^ | Mean change (95% CI)^b^ | n 1^a^ | Mean change (95% CI)^b^ | n 1^a^ | Mean change (95% CI)^b^ |
|  | n 2 | p | n 2 | p | n 2 | p | n 2 | p |
| Weight, kg | 248 | -2.40 (-3.01, -1.79) | 170 | -2.14 (-2.86, -1.42) | 200 | -2.96 (-3.81, -2.11) | 168 | -1.48 (-2.18, -0.77) |
|  | 168 | <0.001 | 119 | <0.001 | 81 | <0.001 | 126 | <0.001 |
| Body Mass Index, kg/m^2^ | 248 | -0.88 (-1.10, -0.66) | 170 | -0.77 (-1.03, -0.51) | 200 | -1.01 (-1.32, -0.70) | 168 | -0.53 (-0.78, -0.28) |
|  | 168 | <0.001 | 119 | <0.001 | 81 | <0.001 | 126 | <0.001 |
| Waist circumference, cm | 246 | -5.03 (-6.09, -3.96) | 170 | -3.28 (-4.55, -2.01) | 199 | -5.59 (-7.17, -4.00) | 166 | -3.80 (-5.07, -2.52) |
|  | 167 | <0.001 | 118 | <0.001 | 73 | <0.001 | 119 | <0.001 |
| MVPA, min/week | 248 | 171 (132, 210) | 170 | 115 (69, 162) | 200 | 159 (103, 214) | 168 | 146 (101, 191) |
|  | 170 | <0.001 | 120 | <0.001 | 81 | <0.001 | 127 | <0.001 |
| Sitting on weekdays, h/day | 247 | -1.07 (-1.46, -0.68) | 170 | -1.33 (-1.80, -0.87) | 199 | -1.30 (-1.82, -0.77) | 166 | -1.06 (-1.52, -0.60) |
|  | 170 | <0.001 | 120 | <0.001 | 81 | <0.001 | 127 | <0.001 |
| Vegetables, serves/day | 248 | 1.08 (0.80, 1.35) | 170 | 1.21 (0.88, 1.54) | 200 | 0.61 (0.23, 0.99) | 168 | 1.09 (0.77, 1.41) |
|  | 170 | <0.001 | 120 | <0.001 | 81 | 0.002 | 127 | <0.001 |
| Fruit, serves/day | 248 | 0.13 (-0.01, 0.27) | 170 | 0.40 (0.23, 0.56) | 200 | 0.35 (0.16, 0.54) | 167 | 0.23 (0.06, 0.39) |
|  | 170 | 0.073 | 120 | <0.001 | 81 | <0.001 | 127 | 0.007 |
| Fat Index, 1–5 | 238 | 0.29 (0.23, 0.36) | 169 | 0.33 (0.26, 0.40) | 199 | 0.29 (0.20, 0.37) | 165 | 0.38 (0.31, 0.45) |
|  | 164 | <0.001 | 120 | <0.001 | 80 | <0.001 | 125 | <0.001 |
| Fibre Index, 1–5 | 238 | 0.20 (0.13, 0.27) | 161 | 0.29 (0.21, 0.38) | 193 | 0.20 (0.10, 0.29) | 161 | 0.25 (0.17, 0.34) |
|  | 159 | <0.001 | 115 | <0.001 | 77 | <0.001 | 110 | <0.001 |
| Physical Quality of Life, 0–100 | 248 | 4.84 (3.34, 6.33) | 170 | 6.68 (4.89, 8.47) | 200 | 6.02 (3.93, 8.12) | 168 | 6.87 (5.12, 8.63) |
|  | 172 | <0.001 | 120 | <0.001 | 81 | <0.001 | 127 | <0.001 |
| Mental Quality of Life, 0–100 | 248 | 1.40 (-0.02, 2.82) | 170 | 3.10 (1.40, 4.80) | 200 | 3.93 (1.95, 5.90) | 168 | 2.20 (0.53, 3.87) |
|  | 172 | 0.054 | 120 | <0.001 | 81 | <0.001 | 127 | 0.010 |
| Symptom Severity, 0–10 | 248 | -0.91 (-1.12, -0.70) | 170 | -0.98 (-1.23, -0.73) | 200 | -0.98 (-1.28, -0.67) | 168 | -1.12 (-1.36, -0.87) |
|  | 171 | <0.001 | 120 | <0.001 | 81 | <0.001 | 127 | <0.001 |
| Symptom Interference, 0–10 | 248 | -1.20 (-1.49, -0.91) | 170 | -1.46 (-1.8, -1.11) | 200 | -1.39 (-1.8, -0.98) | 167 | -1.39 (-1.73, -1.05) |
|  | 171 | <0.001 | 120 | <0.001 | 81 | <0.001 | 127 | <0.001 |
| Fear of Cancer Recurrence, 0–40 | 248 | -3.06 (-4.25, -1.88) | 170 | -4.28 (-5.69, -2.88) | 200 | -3.09 (-4.74, -1.45) | 168 | -3.00 (-4.38, -1.62) |
|  | 170 | <0.001 | 121 | <0.001 | 81 | <0.001 | 127 | <0.001 |
| Distress Level, 0–10 | 246 | -0.82 (-1.21, -0.44) | 170 | -0.74 (-1.20, -0.28) | 200 | -0.56 (-1.09, -0.04) | 168 | -0.71 (-1.16, -0.26) |
|  | 169 | <0.001 | 120 | 0.002 | 81 | 0.035 | 127 | 0.002 |
| Distress Impact, 0–10 | 246 | -0.49 (-0.85, -0.12) | 170 | -0.97 (-1.41, -0.54) | 200 | -0.58 (-1.07, -0.09) | 168 | -0.70 (-1.13, -0.27) |
|  | 169 | 0.009 | 120 | <0.001 | 81 | 0.021 | 127 | 0.001 |

MVPA = moderate-vigorous physical activity

^a^ n 1 = n in model (baseline data); n 2 = in model (post data)

^b^ Change pre to post within Cancer Council estimated by comparison of marginal means; model includes timepoint, Cancer council and timepoint x Cancer Council
