# Supplementary material for: Translating research into practice: outcomes from the Healthy Living after Cancer partnership project
Source: BMC Cancer. 2020 Oct 6;20:963. doi: 10.1186/s12885-020-07454-4 (PMC7539431; doi:10.1186/s12885-020-07454-4)
Supplement: Supplementary file 7 — Additional file 7 : Table 7. Baseline (pre) to post-program changes in patient reported outcomes in Healthy Living after Cancer participants (sensitivity analyses). [file 12885_2020_7454_MOESM7_ESM.docx]

Additional Table 7: Baseline (pre) to post-program changes in patient reported outcomes in Healthy Living after Cancer participants (sensitivity analyses)

| **Outcome** | **Adjusted** | | | | **Multiply imputed (n=786)** | | **Per Protocol (Program Completers)** | | | |
| --- | --- | --- | --- | --- | --- | --- | --- | --- | --- | --- |
|  | **n Pre** | **n Post** | **∆ (95%CI) ^a,b^** | **p** | **∆ (95%CI) ^c^** | **p** | **n Pre** | **n Post** | **∆ (95%CI) ^a^** | **p** |
| Weight, kg | 768 | 480 | -2.30 (-2.66, -1.93) | <0.001 | -2.17 (-2.54, -1.81) | <0.001 | 472 | 472 | -2.24 (-2.63, -1.85) | <0.001 |
| Body Mass Index, kg/m^2^ | 768 | 480 | -0.82 (-0.95, -0.69) | <0.001 | -0.79 (-0.92, -0.66) | <0.001 | 472 | 472 | -0.81 (-0.95, -0.67) | <0.001 |
| Waist circumference, cm | 764 | 764 | -4.44 (-5.11, -3.77) | <0.001 | -4.04 (-5.21, -3.81) | <0.001 | 457 | 458 | -4.32 (-5.00, -3.63) | <0.001 |
| MVPA, min/week | 768 | 484 | 146 (122, 169) | <0.001 | 151 (130, 173) | <0.001 | 476 | 476 | 147 (122, 172) | <0.001 |
| Sitting on weekdays, h/day | 724 | 484 | -1.15 (-1.39, -0.91) | <0.001 | -1.13 (-1.36, -0.91) | <0.001 | 473 | 476 | -1.20 (-1.45, -0.95) | <0.001 |
| Vegetables, serves/day | 768 | 484 | 0.98 (0.81, 1.15) | <0.001 | 1.00 (0.84, 1.16) | <0.001 | 476 | 476 | 0.89 (0.71, 1.08) | <0.001 |
| Fruit, serves/day | 768 | 485 | 0.24 (0.15, 0.32) | <0.001 | 0.24 (0.16, 0.31) | <0.001 | 475 | 476 | 0.24 (0.14, 0.33) | <0.001 |
| Fat Index, 1–5 | 753 | 476 | 0.32 (0.29, 0.36) | <0.001 | 0.32 (0.28, 0.35) | <0.001 | 466 | 468 | 0.32 (0.28, 0.36) | <0.001 |
| Fibre Index, 1–5 | 731 | 448 | 0.22 (0.18, 0.26) | <0.001 | 0.22 (0.18, 0.27) | <0.001 | 433 | 439 | 0.22 (0.18, 0.27) | <0.001 |
| Physical Quality of Life, 0–100 | 768 | 486 | 5.96 (5.04, 6.87) | <0.001 | 5.80 (4.90, 6.70) | <0.001 | 476 | 476 | 6.16 (5.17, 7.15) | <0.001 |
| Mental Quality of Life, 0–100 | 768 | 486 | 2.36 (1.50, 3.22) | <0.001 | 2.59 (1.77, 3.41) | <0.001 | 476 | 476 | 2.35 (1.41, 3.28) | <0.001 |
| Symptom Severity, 0–10 | 768 | 485 | -0.97 (-1.10, -0.84) | <0.001 | -0.97 (-1.09, -0.85) | <0.001 | 476 | 476 | -1.01 (-1.14, -0.87) | <0.001 |
| Symptom Interference, 0–10 | 768 | 499 | -1.32 (-1.49, -1.14) | <0.001 | -1.36 (-1.52, -1.19) | <0.001 | 475 | 476 | -1.31 (-1.50, -1.12) | <0.001 |
| Fear of Cancer Recurrence, 0–40 | 717 | 451 | -3.12 (-3.85, -2.39) | <0.001 | -3.27 (-3.95, -2.60) | <0.001 | 476 | 476 | -3.13 (-3.9, -2.35) | <0.001 |
| Distress Level, 0–10 | 768 | 483 | -0.62 (-0.85, -0.38) | <0.001 | -0.75 (-0.98, -0.52) | <0.001 | 474 | 475 | -0.65 (-0.9, -0.39) | <0.001 |
| Distress Impact, 0–10 | 768 | 483 | -0.66 (-0.88, -0.44) | <0.001 | -0.69 (-0.90, -0.48) | <0.001 | 474 | 475 | -0.55 (-0.79, -0.31) | <0.001 |

MVPA = moderate-vigorous physical activity; CI = confidence interval.

^a^ Pooled mean change estimated by comparison of marginal means for the effect of time (baseline / post program), estimated balanced across strata (Cancer Council: A, B, C, D) from model that includes effects of time, Cancer Council, and their two-way interaction.

^b^ Model also includes predictors of missing data (as listed in Additional File 2).

^c^ Pooled mean change estimated multiply imputed data (m=50 imputations) as effect of time in model that includes effects of time (pre / post) and Cancer Council (A / B / C / D). Variables used in the imputations are shown in Additional File 2.
