# Supplementary material for: Translating research into practice: outcomes from the Healthy Living after Cancer partnership project
Source: BMC Cancer. 2020 Oct 6;20:963. doi: 10.1186/s12885-020-07454-4 (PMC7539431; doi:10.1186/s12885-020-07454-4)
Supplement: Supplementary file 8 — Additional file 8 : Table 8. Patient-reported outcomes in Healthy Living after Cancer program completers by Cancer Council (per-protocol analysis, evaluable cases). [file 12885_2020_7454_MOESM8_ESM.docx]

Additional Table 8: Patient-reported outcomes in Healthy Living after Cancer program completers by Cancer Council (per-protocol analysis, evaluable cases)

| **Outcome** | **A** | | **B** | | **C** | | **D** | |
| --- | --- | --- | --- | --- | --- | --- | --- | --- |
|  | **n 1 ^a^** | **Mean change (95% CI)^b^** | **n 1 ^a^** | **Mean change (95% CI)^b^** | **n 1^a^** | **Mean change (95% CI)^b^** | **n 1^a^** | **Mean change (95% CI)^b^** |
|  | **n 2** | **p** | **n 2** | **p** | **n 2** | **p** | **n 2** | **p** |
| Weight, kg | 163 | -2.40 (-3.03, -1.78) | 113 | -2.16 (-2.91, -1.4) | 70 | -3 (-3.96, -2.04) | 126 | -1.4 (-2.11, -0.69) |
|  | 163 | **<0.001** | 113 | **<0.001** | 70 | **<0.001** | 126 | **<0.001** |
| Body Mass Index, kg/m^2^ | 163 | -0.87 (-1.10, -0.65) | 113 | -0.79 (-1.07, -0.52) | 70 | -1.05 (-1.40, -0.71) | 126 | -0.51 (-0.77, -0.25) |
|  | 163 | **<0.001** | 113 | **<0.001** | 70 | **<0.001** | 126 | **<0.001** |
| Waist circumference, cm | 163 | -4.95 (-6.03, -3.86) | 112 | -3.27 (-4.58, -1.96) | 64 | -5.36 (-7.09, -3.64) | 118 | -3.69 (-4.96, -2.41) |
|  | 163 | **<0.001** | 112 | **<0.001** | 64 | **<0.001** | 119 | **<0.001** |
| MVPA, min/week | 165 | 161.75 (12.32, 202.17) | 114 | 124.69 (76.06, 173.33) | 70 | 153 (91, 215) | 127 | 148 (102, 194) |
|  | 165 | **<0.001** | 114 | **<0.001** | 70 | **<0.001** | 127 | **<0.001** |
| Sitting on weekdays, h/day | 164 | -1.12 (-1.52, -0.71) | 114 | -1.36 (-1.84, -0.87) | 70 | -1.36 (-1.98, -0.73) | 125 | -0.96 (-1.43, -0.5) |
|  | 165 | **<0.001** | 114 | **<0.001** | 70 | **<0.001** | 127 | **<0.001** |
| Vegetables, serves/day | 165 | 1.00 (0.70, 1.30) | 114 | 1.16 (0.80, 1.52) | 70 | 0.41 (-0.04, 0.87) | 127 | 0.99 (0.65, 1.33) |
|  | 165 | **<0.001** | 114 | **<0.001** | 70 | 0.076 | 127 | **<0.001** |
| Fruit, serves/day | 165 | 0.03 (-0.12, 0.18) | 114 | 0.37 (0.18, 0.55) | 70 | 0.30 (0.06, 0.54) | 126 | 0.25 (0.07, 0.42) |
|  | 165 | 0.700 | 114 | **<0.001** | 70 | 0.013 | 127 | 0.007 |
| Fat Index, 1–5 | 159 | 0.29 (0.23, 0.36) | 114 | 0.31 (0.24, 0.39) | 69 | 0.28 (0.18, 0.38) | 124 | 0.37 (0.30, 0.44) |
|  | 160 | **<0.001** | 114 | **<0.001** | 69 | **<0.001** | 125 | **<0.001** |
| Fibre Index, 1–5 | 150 | 0.18 (0.11, 0.26) | 108 | 0.29 (0.20, 0.38) | 66 | 0.18 (0.07, 0.29) | 109 | 0.24 (0.15, 0.32) |
|  | 154 | **<0.001** | 109 | **<0.001** | 66 | 0.002 | 110 | **<0.001** |
| Physical Quality of Life, 0–100 | 165 | 5.16 (3.56, 6.76) | 114 | 6.54 (4.62, 8.46) | 70 | 5.78 (3.33, 8.24) | 127 | 7.15 (5.33, 8.97) |
|  | 165 | **<0.001** | 114 | **<0.001** | 70 | **<0.001** | 127 | **<0.001** |
| Mental Quality of Life, 0–100 | 165 | 1.22 (-0.30, 2.73) | 114 | 2.51 (0.68, 4.33) | 70 | 3.95 (1.62, 6.27) | 127 | 1.73 (0.01, 3.46) |
|  | 165 | 0.115 | 114 | 0.007 | 70 | **<0.001** | 127 | 0.049 |
| Symptom Severity, 0–10 | 165 | -0.93 (-1.14, -0.71) | 114 | -0.94 (-1.20, -0.69) | 70 | -1.06 (-1.39, -0.73) | 126 | -1.09 (-1.34, -0.85) |
|  | 165 | **<0.001** | 114 | **<0.001** | 70 | **<0.001** | 127 | **<0.001** |
| Symptom Interference, 0–10 | 165 | -1.20 (-1.50, -0.90) | 114 | -1.26 (-1.63, -0.90) | 70 | -1.46 (-1.92, -0.99) | 126 | -1.32 (-1.66, -0.98) |
|  | 165 | **<0.001** | 114 | **<0.001** | 70 | **<0.001** | 127 | **<0.001** |
| Fear of Cancer Recurrence, 0–40 | 165 | -3.03 (-4.29, -1.77) | 114 | -3.87 (-5.38, -2.35) | 70 | -2.79 (-4.72, -0.85) | 127 | -2.82 (-4.26, -1.38) |
|  | 165 | **<0.001** | 114 | **<0.001** | 70 | 0.005 | 127 | **<0.001** |
| Distress Level, 0–10 | 163 | -0.81 (-1.23, -0.40) | 114 | -0.54 (-1.04, -0.05) | 70 | -0.61 (-1.25, 0.02) | 127 | -0.61 (-1.08, -0.14) |
|  | 164 | **<0.001** | 114 | 0.032 | 70 | 0.058 | 127 | 0.012 |
| Distress Impact, 0–10 | 163 | -0.41 (-0.80, -0.02) | 114 | -0.64 (-1.11, -0.17) | 70 | -0.64 (-1.24, -0.05) | 127 | -0.51 (-0.95, -0.07) |
|  | 164 | 0.039 | 114 | 0.007 | 70 | 0.035 | 127 | 0.024 |

MVPA = moderate-vigorous physical activity

^a^ n 1 = n in model (baseline data); n 2 = in model (post data)

^b^ Change pre to post within Cancer Council estimated by comparison of marginal means; model includes timepoint, Cancer Council and timepoint x Cancer Council
